# Delivering improved survivorship care for people affected by advanced or metastatic cancer

**DOI:** 10.2340/1651-226X.2024.42197

**Published:** 2024-12-09

**Authors:** Julia Lai-Kwon, Sarah Heynemann, Nicolas H. Hart, Raymond J. Chan, Thomas J. Smith, Andrea L. Smith, Larissa Nekhlyudov, Michael Jefford

**Affiliations:** a Department of Medical Oncology, Peter MacCallum Cancer Centre, Melbourne, VIC, Australia; b Department of Health Services Research, Peter MacCallum Cancer Centre, Melbourne, VIC, Australia; c Sydney Health Ethics, Faculty of Medicine and Health, The University of Sydney, Sydney, NSW, Australia; d Caring Futures Institute, College of Nursing and Health Sciences, Flinders University, Adelaide, SA, Australia; e Human Performance Research Centre, INSIGHT Research Institute, Faculty of Health, University of Technology Sydney (UTS), Sydney, NSW, Australia; f Exercise Medicine Research Institute, School of Medical and Health Science, Edith Cowan University, Perth, WA, Australia; g Institute for Health Research, The University of Notre Dame Australia, Perth, WA, Australia; h Cancer and Palliative Care Outcomes Centre, Faculty of Health, Queensland University of Technology (QUT), Brisbane, QLD, Australia; i Division of General Internal Medicine, Section of Palliative Medicine, Johns Hopkins Medical Institutions, New York, NY, USA; j Sidney Kimmel Comprehensive Cancer Centre, Johns Hopkins Hospital, Baltimore, MD, USA; k The Daffodil Centre, The University of Sydney, a joint venture with Cancer Council, Sydney, Australia; l Brigham and Women’s Hospital, Harvard Medical School, Boston, MA, USA; m Australian Cancer Survivorship Centre, Peter MacCallum Cancer Centre, Melbourne, VIC, Australia; n Sir Peter MacCallum Department of Oncology, University of Melbourne, Melbourne, VIC, Australia

**Keywords:** Advanced cancer, metastatic cancer, survivorship, supportive care

## Introduction to advanced or metastatic cancer survivorship

There is a growing population of people living long term with advanced or metastatic cancer [1, 2]. Though they are technically considered to be ‘cancer survivors’ according to the widely accepted definition of survivorship as beginning at the time of diagnosis and spanning the balance of life [3], historically, many people had a poor prognosis with limited survival. Consequently, the focus of their care was symptom palliation and end-of-life care. Survivorship was therefore not a clinical, research, or policy term applied to or embraced by this population.

**Figure 1:**
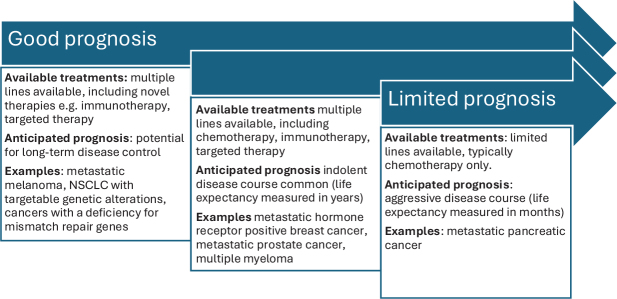
Variable disease trajectories of people living with advanced or metastatic cancer [6]. NSCLC: Non-Small Cell Lung Cancer.

Owing to the earlier detection of metastatic disease, treatment and supportive care advances, a growing number of people with advanced or metastatic cancer are now living longer [1, 2], have supportive needs beyond palliation, and may therefore experience cancer more akin to a chronic disease. It is important to acknowledge that the term ‘cancer survivor’ may not resonate with all people living with advanced or metastatic cancer [4, 5]. This is a diverse population with considerable heterogeneity in terms of disease characteristics (tumour type, number and site of metastases), treatment modalities (type and number available), and disease trajectory (indolent vs. ‘aggressive’) (Figure 1). Accordingly, we must shift the survivorship care paradigm to make it relevant to people living with advanced or metastatic cancer [6].

## What issues are faced by people affected by advanced or metastatic cancer?

While some issues experienced by people with advanced or metastatic cancer are similar to those experienced by survivors of early-stage cancers, others may be distinct or heightened [7, 8]. These include complex toxicity profiles (high grade toxicities from multimodal treatments and/or repeated, cumulative low grade toxicities), psychological burden (e.g. fear of cancer progression, prognostic uncertainty), barriers to accessing insurance or disability support, difficulties with continuing or returning to work, and subsequent financial stress. Over the course of their disease trajectory, people with advanced or metastatic cancer may experience more frequent, complex, and longer-term healthcare system engagements than those with early-stage cancers [6, 9]. Caregivers also experience unique challenges compared to those caring for people with early-stage cancer related to the extended duration of illness, uncertain disease trajectory and prognosis, complex treatment decisions [7], ongoing financial stress and compassion fatigue.

## Improving survivorship care for people living with advanced or metastatic cancer

Several groups have defined the key components of high-quality advanced and metastatic survivorship care [6, 10], drawing upon the principles of quality cancer survivorship care [11]. These components include screening for and responding to supportive care needs, providing coordinated, multidisciplinary care that is evidence-based and culturally appropriate, ensuring care is accessible and equitable, and adequately resourced and evaluated [6, 10]. However, current survivorship care for people with advanced or metastatic cancer does not meet these standards. This is in part because their needs are not routinely considered, or prioritised by, survivorship care services providers. Consequently, appropriate services designed for their specific needs often do not exist. In this commentary, we outline key actions needed in clinical practice to improve survivorship care for people living with advanced or metastatic cancer, emphasising the real-world challenges of care delivery and possible strategies to mitigate these challenges.

1. Clear, regular discussions regarding prognosis

People with advanced or metastatic cancer and their caregivers should be provided with tailored and realistic prognostic information to help set rational expectations and facilitate informed decision-making throughout their disease trajectory [12, 13]. Ensuring discussions about prognosis are linked with discussions regarding a person’s life goals and goals of care will help to ensure personal agency is respected and supported through shared decision making [10]. Communicating about prognosis with primary care professionals is also critical to inform appropriate non-cancer-related care (such as screening for other cancers, management of chronic medical conditions, and health promotion) [11]. Other healthcare professionals, such as medical specialists and rehabilitation clinicians, would also benefit from prognostic information. If prognosis is deemed poor, early involvement of palliative/end-of-life clinicians would be appropriate.

We acknowledge that there are challenges in discussing prognosis, including the inherent uncertainty of an individual’s disease course, maintaining hope while avoiding unrealistic optimism or nihilism (‘hoping for the best while planning for the rest’), variable education and awareness among non-speciality providers of cancer care of therapeutic and prognostic advances, and management of misinformation in mainstream and social media. Existing guidelines [14] and tools [15] may assist such discussions.

2. Proactive assessment of physical and psychosocial unmet needs and referral to appropriate supportive care services

Hart and colleagues [10] have emphasised the need for person-centred care for people with advanced or metastatic cancer that addresses their unique physical, psychological, informational, financial, fertility, sexual, and spiritual needs in a humane and holistic manner. In clinical settings, screening for physical and psychosocial needs should be proactive and ideally utilise validated patient-reported outcome measures (PROMs) followed by conversations with appropriate healthcare professionals to address these needs [16, 17]. Screening should be conducted at diagnosis and at regular time points throughout the disease trajectory (e.g. following a change in treatment or transition to palliative/end-of-life care).

Based on the needs identified, early referral to multidisciplinary supportive care services should be initiated. While services and resources should be tailored to the specific needs of this population, accessible (i.e. affordable, acceptable, available and appropriate) [10] services and resources may not be routinely available. Some health behaviour or lifestyle interventions could be delivered by non-government organisations, in community settings [18], or via telehealth [19]. Self-management strategies should be provided where possible, with consideration of the individual’s health literacy [20]. Peer support could also be included [10, 21].

The above recommendations, including the implementation of PROMs, clinical intervention and/or referral to resources and services, provide opportunities to improve supportive care for people affected by advanced or metastatic cancer. Strategies could include creating stepped care models to better allocate resources. For example, those with lower levels of unmet need or higher levels of health literacy could be supported to self-manage [20], including referral to support groups [21], peer support [22], allowing telemedicine instead of in-person visits [23], and ‘stepped’ access to palliative care services (initial visits, then visits as needed instead of as per a standardised schedule [24]). Services delivered by cancer centres could then be reserved for those with the greatest level of unmet need. Non-government organisations and community groups could also be integrated into referral pathways to provide supportive care services to those with less complex needs [18].

3. Create novel models of care blending elements of survivorship and palliative care services

Both survivorship and palliative care focus on delivering person-centred care and facilitating shared decision making. However, survivorship care focuses on managing longer-term physical and psychosocial issues, comorbidities, and surveillance for new malignancies, whereas palliative care is typically directed towards management of acute physical and psychosocial needs, prognostic discussions, and management of spiritual and existential needs [25–27]. Early access to palliative care is a critical component of comprehensive cancer care for many people living with advanced or metastatic cancer [28]. New models of advanced and metastatic survivorship care should include elements of both, focusing on equitable access to appropriate services specifically designed to meet the unique needs of people with advanced or metastatic cancer and their caregivers, such as specialist advanced or metastatic survivorship nurses [29, 30] and peer support [21].

Challenges to achieving blended palliative/survivorship care may be related to differing patient and healthcare provider expectations for both types of care. For example, people with advanced or metastatic cancer may not identify with a ‘survivorship program’ or find a ‘palliative care’ relevant while living with cancer, while healthcare providers may not fully understand the specific needs of this emerging population. Both programmes have challenges in staffing, funding and other institutional barriers. Potential strategies could include advocacy to highlight the survivorship needs of those with advanced or metastatic cancer, clearly defining the goals of novel models of care, encouraging endorsement of these models of care by the treating oncology teams, and possibly rebranding models of care to ‘enhanced supportive care’ [31].

4. Improve communication and coordination of care between healthcare providers and settings

People with advanced or metastatic cancer often experience multiple care transitions between community and hospital-based healthcare settings and are treated by multiple healthcare providers. Ensuring continuity of care by integrating health services (e.g. medical specialists, nursing, primary care, and allied health) across the continuum of care is therefore a priority [32]. Challenges include poorly coordinated and often siloed healthcare disciplines and systems, a lack of clarity between healthcare professionals about roles and responsibilities, people lacking the proficiency or health literacy to navigate the healthcare system [33], and a lack of access to shared electronic medical records. Strategies may include clear documentation of a person’s diagnosis, prognosis, goals of care, and management. Such communication may occur in a survivorship care plan [34], advanced care plan [35] or any structured tool used in clinical settings, including a clinical note. Patient navigation services [33], peer support [21], and empowering those with advanced or metastatic cancer and their caregivers to assist with care coordination may also assist.

## Conclusion

People with advanced or metastatic cancer and their caregivers face a range of physical, psychosocial, and informational concerns and unmet needs. Current care systems operate in a resource-constrained environment and are not organised to systematically screen for, identify and address these needs. The past decade has resulted in dramatic changes in the treatment, prognosis and numbers of those living with advanced or metastatic cancer and has led to a critical need to reimagine and reorganise care to ensure optimal survivorship care is provided with available resources. This will require a context-specific, flexible approach integrating survivorship, palliative, primary, and community-based care services that is responsive to the individual’s disease trajectory and needs.

## Data Availability

No participant data is reported in this article.

## References

[1] Gallicchio L, Devasia TP, Tonorezos E, Mollica MA, Mariotto A. Estimation of the numbers of individuals living with metastatic cancer in the United States. J Natl Cancer Inst. 2022;114(11):djac158. 10.1093/jnci/djac158 PMC9949565 35993614

[2] White R, Stanley F, Than J, Macnair A, Pethick J, Fallica G, et al. Treatable but not curable cancer in England: a retrospective cohort study using cancer registry data and linked data sets. BMJ Open. 2021;11(1):e040808. 10.1136/bmjopen-2020-040808 PMC7798682 33419907

[3] National Coalition for Cancer Survivorship. NCCS mission 2017 [cited 2017 May 26]. Available from: https://www.canceradvocacy.org/about-us/our-mission/.

[4] Mollica MA, Smith AW, Tonorezos E, Castro K, Filipski KK, Guida J, et al. Survivorship for individuals living with advanced and metastatic cancers: National Cancer Institute Meeting Report. J Natl Cancer Inst. 2021;114(4):489–95. 10.1093/jnci/djab223 PMC9002286 34878107

[5] Smith AL, Hart NH, Jefford M, Chan RJ. Survivorship research for people with metastatic or advanced cancer: a time for action. Asia Pac J Oncol Nurs. 2022;9(4): 185–6. 10.1016/j.apjon.2022.02.001 35571628 PMC9096729

[6] Lai-Kwon J, Heynemann S, Hart NH, Chan RJ, Smith TJ, Nekhlyudov L, et al. Evolving landscape of metastatic cancer survivorship-reconsidering clinical care, policy, and research priorities for the modern era. J Clin Oncol. 2023:Jco2202212. 10.1200/JCO.22.02212 36848622

[7] Hart NH, Crawford-Williams F, Crichton M, Yee J, Smith TJ, Koczwara B, et al. Unmet supportive care needs of people with advanced cancer and their caregivers: a systematic scoping review. Crit Rev Oncol Hematol. 2022;176:103728. 10.1016/j.critrevonc.2022.103728 35662585

[8] Wang T, Molassiotis A, Chung BPM, Tan J-Y. Unmet care needs of advanced cancer patients and their informal caregivers: a systematic review. BMC Palliat Care. 2018;17(1):96. 10.1186/s12904-018-0346-9 30037346 PMC6057056

[9] Jacobsen PB, Nipp RD, Ganz PA. Addressing the survivorship care needs of patients receiving extended cancer treatment. Am Soc Clin Oncol Educ Book. 2017;37:674–83. 10.1200/EDBK_175673 28561717

[10] Hart NH, Nekhlyudov L, Smith TJ, Yee J, Fitch MI, Crawford GB, et al. Survivorship care for people affected by advanced or metastatic cancer: MASCC-ASCO standards and practice recommendations. JCO Oncol Pract. 2024;32. 10.1007/s00520-024-08465-8 38684036

[11] Nekhlyudov L, Mollica MA, Jacobsen PB, Mayer DK, Shulman LN, Geiger AM. Developing a quality of cancer survivorship care framework: implications for clinical care, research, and policy. J Natl Cancer Inst. 2019;111(11):1120–30. 10.1093/jnci/djz089 31095326 PMC6855988

[12] Moth EB, Stockler MR, Kiely BE. Estimating prognosis in advanced cancer: time to embrace uncertainty. J Geriatr Oncol. 2022;13(3):389–90. 10.1016/j.jgo.2021.11.014 34896060

[13] Vasista A, Stockler MR, Martin A, Lawrence NJ, Kiely BE. Communicating prognostic information: what do oncologists think patients with incurable cancer should be told? Intern Med J. 2020;50(12):1492–9. 10.1111/imj.14739 31904887

[14] Gilligan T, Coyle N, Frankel RM, Berry DL, Bohlke K, Epstein RM, et al. Patient-clinician communication: American Society of Clinical Oncology Consensus Guideline. J Clin Oncol. 2017;35(31):3618–32. 10.1200/JCO.2017.75.2311 28892432

[15] Kiely BE, Stockler MR. Discussing prognosis, preferences, and end-of-life care in advanced cancer: we need to speak. JAMA Oncol. 2019;5(6):788–9. 10.1001/jamaoncol.2019.0291 30870565

[16] Presley CJ, Arrato NA, Shields PG, Carbone DP, Wong ML, Benedict J, et al. Functional trajectories and resilience among adults with advanced lung cancer. JTO Clin Res Rep. 2022;3(6):100334. 10.1016/j.jtocrr.2022.100334 35719868 PMC9198463

[17] Le Boutillier C, Jeyasingh-Jacob J, Jones L, King A, Archer S, Urch C. Improving personalised care and support planning for people living with treatable-but-not-curable cancer. BMJ Open Qual. 2023;12(3): e002322. 10.1136/bmjoq-2023-002322 PMC10481844 37666580

[18] Brick RS, Gallicchio L, Mollica MA, Zaleta AK, Tonorezos ES, Jacobsen PB, et al. Survivorship concerns among individuals diagnosed with metastatic cancer: findings from the Cancer Experience Registry. J Cancer Surviv. 2024 (published online 9/4/24). 10.1007/s11764-024-01573-8 38592607

[19] Jones JM, Saeed H, Katz MS, Lustberg MB, Forster VJ, Nekhlyudov L. Readdressing the needs of cancer survivors during COVID-19: a path forward. J Natl Cancer Inst. 2021;113(8):955–61. 10.1093/jnci/djaa200 33367655 PMC7799033

[20] Howell D, Mayer DK, Fielding R, Eicher M, Verdonck-de Leeuw IM, Johansen C, et al. Management of cancer and health after the clinic visit: a call to action for self-management in cancer care. J Natl Cancer Inst. 2021;113(5):523–31. 10.1093/jnci/djaa083 32525530 PMC8096367

[21] Li Z, Laginha K-J, Boyle F, Daly M, Dinner F, Hirsch P, et al. Professionally led support groups for people living with advanced or metastatic cancer: a systematic scoping review of effectiveness and factors critical to implementation success within real-world healthcare and community settings. J Cancer Surviv. 2024 (published online 8/1/24). 10.1007/s11764-023-01515-w PMC12081543 38191752

[22] Walshe C, Roberts D. Peer support for people with advanced cancer: a systematically constructed scoping review of quantitative and qualitative evidence. Curr Opin Support Palliat Care. 2018;12(3):308–22. 10.1097/SPC.0000000000000370 29979318

[23] Greer JA, Temel JS, El-Jawahri A, Rinaldi S, Kamdar M, Park ER, et al. Telehealth vs in-person early palliative care for patients with advanced lung cancer: a multisite randomized clinical trial. JAMA. 2024;332(14):1153–64. 10.1001/jama.2024.13964 39259563 PMC11391365

[24] Temel JS, Jackson VA, El-Jawahri A, Rinaldi SP, Petrillo LA, Kumar P, et al. Stepped palliative care for patients with advanced lung cancer: a randomized clinical trial. JAMA. 2024;332(6):471–81. 10.1001/jama.2024.10398 38824442 PMC11145511

[25] Sedhom R, Bates-Pappas GE, Feldman J, Elk R, Gupta A, Fisch MJ, et al. Tumor is not the only target: ensuring equitable person-centered supportive care in the era of precision medicine. Am Soc Clin Oncol Educ Book. 2024;44(3):e434026. 10.1200/EDBK_434026 39177644

[26] Geerse OP, Lakin JR, Berendsen AJ, Alfano CM, Nekhlyudov L. Cancer survivorship and palliative care: shared progress, challenges, and opportunities. Cancer. 2018;124(23):4435–41. 10.1002/cncr.31723 30204237

[27] Stegmann ME, Geerse OP, van Zuylen L, Nekhlyudov L, Brandenbarg D. Improving care for patients living with prolonged incurable cancer. Cancers (Basel). 2021;13(11):2555. 10.3390/cancers13112555 34070954 PMC8196984

[28] Ferrell BR, Temel JS, Temin S, Alesi ER, Balboni TA, Basch EM, et al. Integration of palliative care into standard oncology care: American Society of Clinical Oncology Clinical Practice Guideline Update. J Clin Oncol. 2017;35(1):96–112. 10.1200/JCO.2016.70.1474 28034065

[29] Warren M, Mackie D, Leary A. The complexity of non face-to-face work with patients affected by metastatic breast cancer and their carers. The ‘hidden consultations’ of the clinical nurse specialist. Eur J Oncol Nurs. 2012;16(5):460–4. 10.1016/j.ejon.2011.10.009 22154555

[30] Lai-Kwon J, Kelly B, Lane S, Biviano R, Bartula I, Brennan F, et al. Feasibility, acceptability, and utility of a nurse-led survivorship program for people with metastatic melanoma (MELCARE). Support Care Cancer. 2022;30(11):9587–96. 10.1007/s00520-022-07360-4 36136246 PMC9492451

[31] Berman R, Davies A. Enhanced supportive care is broader than palliative care. BMJ. 2019;365:l1629. 10.1136/bmj.l1629 30971400

[32] Nekhlyudov L, Birken SA, Mayer DK. Living with advanced cancer and the role of the primary care provider: the missing piece in the survivorship discourse. Eur J Cancer Care (Engl). 2017;26(3). 10.1111/ecc.12708 28497519

[33] Chan RJ, Milch VE, Crawford-Williams F, Agbejule OA, Joseph R, Johal J, et al. Patient navigation across the cancer care continuum: an overview of systematic reviews and emerging literature. CA Cancer J Clin. 2023;73(6):565–89. 10.3322/caac.21788 37358040

[34] Mayer DK, Green M, Check DK, Gerstel A, Chen RC, Asher G, et al. Is there a role for survivorship care plans in advanced cancer? Support Care Cancer. 2015;23(8):2225–30. 10.1007/s00520-014-2586-4 25559037

[35] Levy D, Dhillon HM, Lomax A, Marthick M, McNeil C, Kao S, et al. Certainty within uncertainty: a qualitative study of the experience of metastatic melanoma patients undergoing pembrolizumab immunotherapy. Support Care Cancer. 2019;27(5):1845–52. 10.1007/s00520-018-4443-3 30178142

